# Case Report: Carotid cavernous fistula presenting as red eye: case illustration and comprehensive review

**DOI:** 10.3389/fmed.2025.1613326

**Published:** 2025-07-21

**Authors:** Qi-Bin Xu, Wen-Yan Sheng

**Affiliations:** Department of Ophthalmology, Zhejiang Hospital of Integrated Traditional Chinese and Western Medicine, Hangzhou Red-Cross Hospital, Hangzhou, China

**Keywords:** carotid cavernous fistula, red eye, ophthalmic manifestations, diagnosis, treatment

## Abstract

Carotid cavernous fistula (CCF) is a pathological condition where an abnormal connection forms between the carotid artery and the cavernous sinus. This condition can lead to a variety of ocular and neurological symptoms, often making it difficult to diagnose, especially when patients present with common ocular complaints such as red eye, which is often misdiagnosed as conjunctivitis. The subtle and diverse symptoms of CCF can lead to misdiagnosis, delaying appropriate treatment. This article reports a case of CCF that initially presented with red eye and proptosis, leading to an ophthalmology referral. By comparing pre- and postoperative ocular photographs, the article provides a detailed review of the ophthalmic features, classification, diagnostic methods, and treatment options for CCF. The aim is to enhance clinicians’ understanding of this condition, improve diagnostic accuracy at initial presentation, and promote timely and effective treatment.

## 1 Introduction

Carotid cavernous fistula (CCF) is a pathological condition where an abnormal connection forms between the carotid artery and the cavernous sinus. This condition can lead to a variety of ocular and neurological symptoms, often making it difficult to diagnose, especially when patients present with common ocular complaints such as red eye, which is often misdiagnosed as conjunctivitis. The subtle and diverse symptoms of CCF can lead to misdiagnosis, delaying appropriate treatment. This article reports a case of CCF that initially presented with red eye and proptosis, leading to an ophthalmology referral. By comparing pre- and postoperative ocular photographs, the article provides a detailed review of the ophthalmic features, classification, diagnostic methods, and treatment options for CCF. The aim is to enhance clinicians’ understanding of this condition, improve diagnostic accuracy at initial presentation, and promote timely and effective treatment.

## 2 Case report

**Figure 1:**
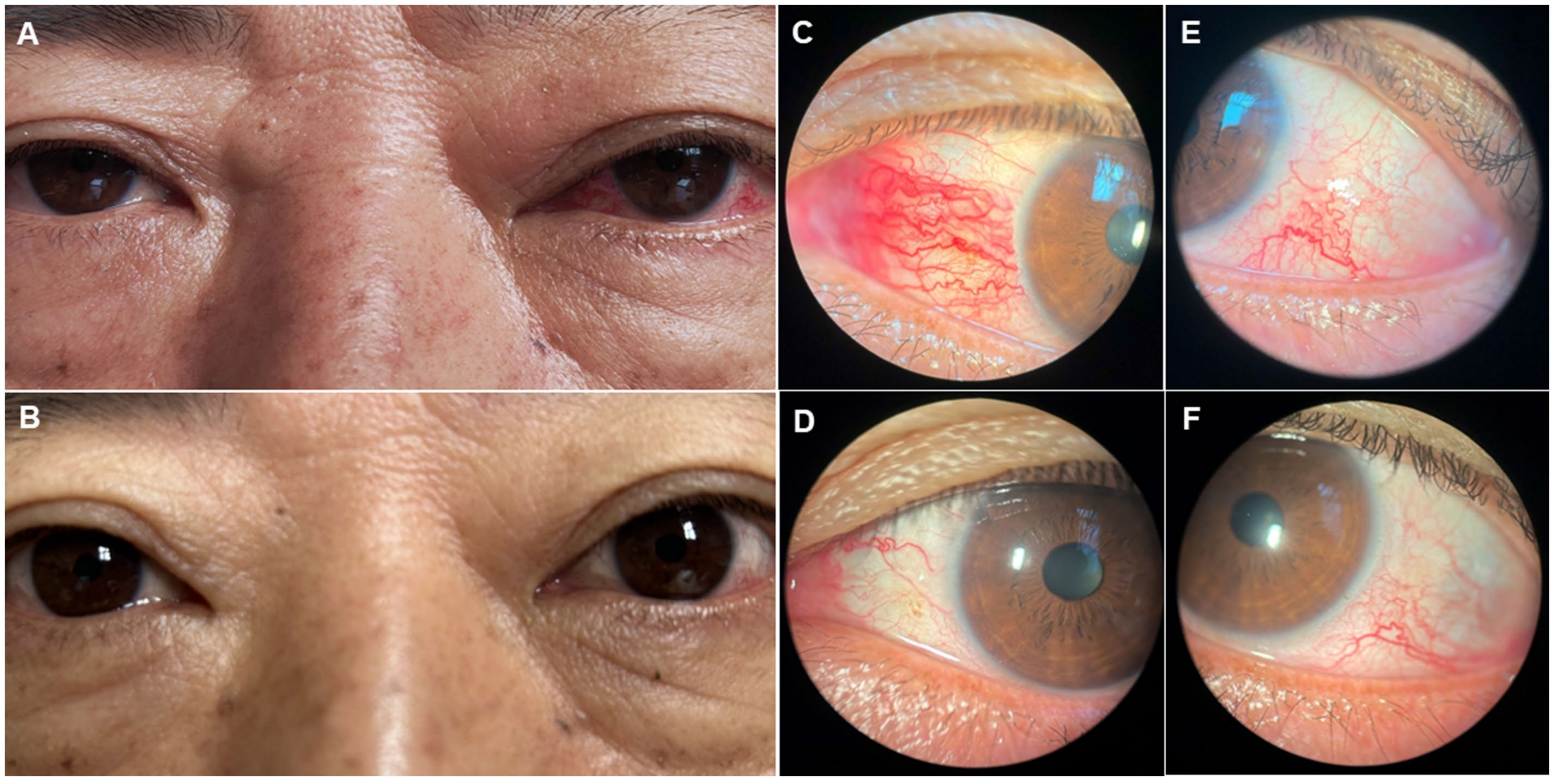
Figures **A** and **B** show clinical photos of the patient’s eyes before and after surgical treatment for left carotid cavernous sinus fistula. Figures **C** and **D** show the dilation and regression of the left eye nasal conjunctival blood vessels before and after surgery. Figures **E** and **F** show the dilation and regression of left temporal conjunctival blood vessels before and after surgery.

**Figure 2:**
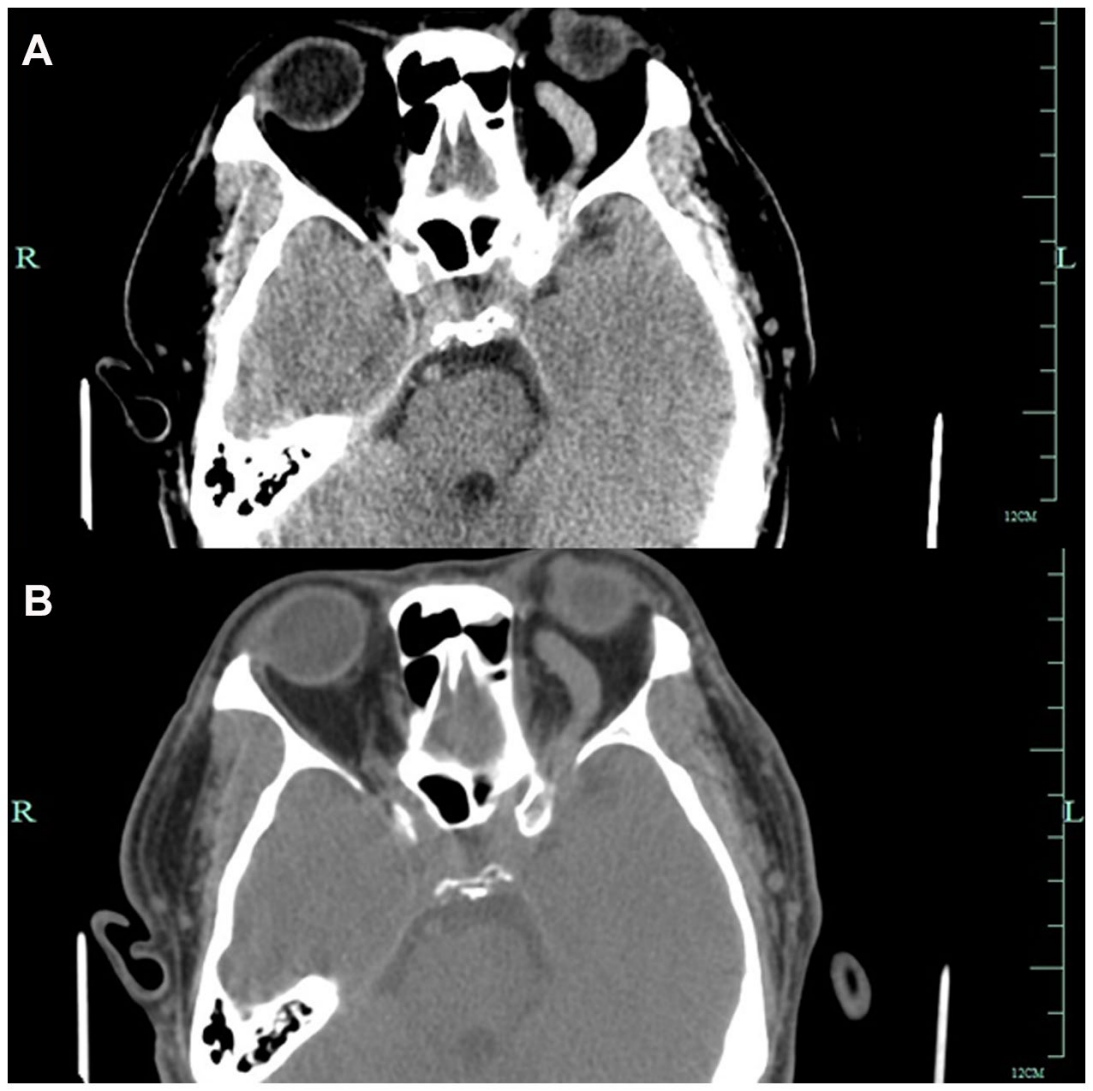
Figures **A** and **B** show Axial CT images showing left-sided proptosis with thickening of the sclera. The left optic foramen is enlarged, and the left cavernous sinus exhibits heterogeneous density. The left superior orbital vessels appear tortuous and dilated.

A 52-year-old man presented to the ophthalmology department with a 3-month history of left eye redness and proptosis (Figure 1A). The patient reported no history of trauma or systemic diseases. Ophthalmic examination revealed significant tortuosity and dilation of conjunctival veins, particularly prominent in the nasal area (Figures 1C,E). Mild left eye proptosis was noted, but visual acuity and ocular motility were relatively intact. Intraocular pressure was within normal limits. Given the clinical findings, a cranial computed tomography was performed, which suggested a suspected a left carotid cavernous sinus fistula (Figures 2A,B). The patient was subsequently referred to the neurosurgery department for further management.

The patient was diagnosed with carotid cavernous sinus fistula. Subsequently, the patient underwent endovascular embolization, during which a detachable balloon was used to occlude the fistula. Post-procedure, the patient’s left eye proptosis improved significantly, and the conjunctival dilation and edema were notably alleviated (Figures 1B,D,F). Follow-up examinations at 1 month and 3 months post-treatment showed no recurrence of symptoms, and the patient remained asymptomatic.

## 3 Discussion

### 3.1 Clinical manifestations of CCF

CCF can present with a wide array of ocular symptoms, making it a diagnostic challenge. Common manifestations include conjunctival chemosis and congestion, proptosis, ocular motility impairments, and visual disturbances. In the presented case, the patient’s red eye and conjunctival vascular changes were typical early manifestations of CCF. Preechawat et al. (1) reported that a significant proportion of CCF patients present with multiple concurrent signs, such as proptosis in 84%, tortuous and dilated conjunctival veins in 93%, chemosis in 42%, cranial nerve palsy and related disorders in 52%, elevated intraocular pressure in 51%, optic neuropathy in 13%, and visual impairment in 43%. These data underscore the importance of a comprehensive ocular assessment in suspecting CCF, especially in patients with unexplained or persistent ocular symptoms.

### 3.2 Diagnosis of CCF

#### 3.2.1 Imaging modalities

Imaging plays a crucial role in the diagnosis of CCF. Orbital color Doppler ultrasound is a non-invasive imaging modality that can detect dilated superior ophthalmic veins and thickened extraocular muscles. It can also observe the characteristic pulsation of the veins synchronous with the cardiac cycle, providing direct evidence of arterial blood flow into the venous system. Computed tomography (CT) and magnetic resonance imaging (MRI) are also important tools in CCF diagnosis (2), as they can reveal structural changes such as enlargement of the cavernous sinus and proptosis. However, digital subtraction angiography (DSA) remains the gold standard for CCF diagnosis. DSA can precisely localize the position, size, and vascular connections of the fistula, thereby guiding subsequent therapeutic strategies (3).

#### 3.2.2 Differential diagnosis

The symptoms of CCF often mimic those of other ocular diseases, leading to potential misdiagnosis. Conditions such as conjunctivitis (4), thyroid eye disease, orbital inflammatory syndrome, and glaucoma can present with similar symptoms (5). Therefore, a meticulous medical history, comprehensive physical examination, and multimodal imaging analysis are essential to differentiate CCF from these conditions (6). For instance, in patients with suspected CCF, the presence of conjunctival vascular tortuosity and dilation, especially when accompanied by other ocular signs or abnormal imaging findings, should prompt further investigation for CCF.

### 3.3 Treatment of CCF

The treatment approach for CCF varies depending on factors such as the size and flow rate of the fistula, as well as the patient’s symptoms. Conservative management, including carotid compression or medication, may be considered for low-flow, asymptomatic fistulas to promote thrombosis and relieve symptoms (7). However, most high-flow fistulas require interventional treatment. With the advancement of endovascular techniques, embolization using coils, balloons, or liquid embolic agents has become the primary treatment modality, achieving high occlusion rates and favorable outcomes (8, 9). In some cases, surgical decompression may be necessary when complications persist after embolization.

## 4 Conclusion

CCF presenting with red eye can be easily overlooked or misdiagnosed, leading to delayed treatment and potential complications. Clinicians should maintain a high index of suspicion when encountering patients with unexplained ocular symptoms, especially those with persistent red eye accompanied by other ocular abnormalities. Timely utilization of appropriate imaging modalities and referral for further evaluation can significantly enhance diagnostic accuracy and ensure prompt treatment, thereby preventing potential visual impairment and neurological complications. Through this case report and comprehensive review, we aim to raise clinicians’ awareness and understanding of CCF, facilitating early and accurate diagnosis in clinical practice.

## Data Availability

The original contributions presented in the study are included in the article/Supplementary material, further inquiries can be directed to the corresponding author/s.
