# Supplementary figures and images for: Case Report: Carotid cavernous fistula presenting as red eye: case illustration and comprehensive review
Source: Front Med (Lausanne). 2025 Jul 21;12:1613326. doi: 10.3389/fmed.2025.1613326 (PMC12318932; doi:10.3389/fmed.2025.1613326)

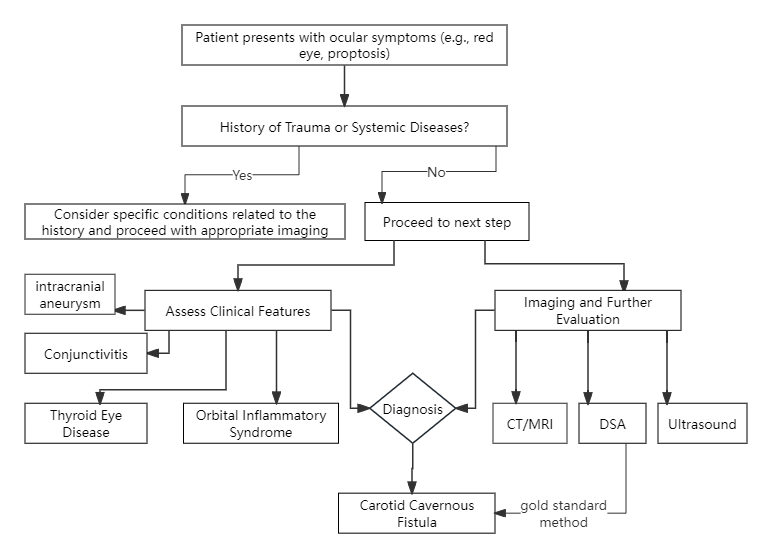

Supplement: Supplementary file 1 [file Image_1.png]
